# Lipocalin 2 in the central nervous system host response to systemic lipopolysaccharide administration

**DOI:** 10.1186/1742-2094-8-124

**Published:** 2011-09-26

**Authors:** Jacque PK Ip, Aline L Noçon, Markus J Hofer, Sue Ling Lim, Marcus Müller, Iain L Campbell

**Affiliations:** 1 Division of Life Science, Department of Neuroscience, The Hong Kong University of Science and Technology, Clear Water Bay, Kowloon, Hong Kong, China; 2 School of Molecular Bioscience and Bosch Institute, Building G08, The University of Sydney, Sydney N.S.W., 2006, Australia; 3 Department of Neuropathology, University Clinic of Marburg and Giessen, Baldingerstraße D-35033, Marburg, Germany; 4 Department of Neurology, The University of Bonn, Sigmund-Freud-Straße 25, 53105 Bonn, Germany

## Abstract

**Background:**

Lipocalin 2 (Lcn2) is a bacteriostatic factor that may also modulate cellular function, however, little is known concerning the expression or role of Lcn2 in CNS inflammation. Therefore, here we investigated the regulation and possible function of Lcn2 in the CNS following peripheral lipopolysaccharide (LPS) injection in mice.

**Methods:**

A murine model for systemic endotoxemia was used in this study. Wild type or Lcn2 KO mice (both genotypes C57BL/6 strain) were given either a single or dual, staggered intraperitoneal injections of purified *E. coli *LPS or vehicle alone. The brain was examined for the expression and location of Lcn2 mRNA and protein and various markers for neuroinflammation were analyzed.

**Results:**

Although undetectable under physiological conditions, both Lcn2 mRNA and protein were induced to high levels in the brain after LPS injection. By contrast, RNA corresponding to the putative Lcn2 (termed 24p3R) receptor was present at high levels in the normal brain and remained unaltered by LPS injection. Differences between Lcn2 and 24p3R mRNA expression were found at the anatomic and cellular level. Endothelial cells, microglia and the choroid plexus but not neurons were identified as the main cellular sources for Lcn2 mRNA in the CNS. By contrast, 24p3R mRNA was detected in neurons and the choroid plexus only. Lcn2 protein was found to have a similar cellular localization as the corresponding RNA transcripts with the exception that subsets of neurons were also strongly positive. Various inflammatory, glial, and iron handling markers were analyzed and found to have similar alterations between WT and Lcn2 KO animals.

**Conclusions:**

1) Lcn2 production is strongly induced in the CNS by systemic LPS injection, 2) in addition to Lcn2 production at key gateways of bacterial entry to the CNS, neurons may be a target for the actions of Lcn2, which is apparently taken up by these cells, and 3) the cellular functions of Lcn2 in the CNS remain enigmatic.

## Background

Lipocalin 2 (Lcn2; also known in humans as neutrophil gelatinase-associated lipocalin (NGAL) [1] and in the mouse as 24p3 [2]) is a small, secreted protein that belongs to the lipocalin family which comprises over 20 members [3]. Despite the low sequence homology (around 20%), all lipocalin family members share a common tertiary structure, namely an eight-stranded, continuously hydrogen bonded anti-parallel ß-barrel [3]. Whereas most lipocalins bind to lipophilic molecules, Lcn2 binds to a class of bacterial metal-binding molecules called siderophores [4].

Lcn2 is involved in anti-microbial defence by sequestering iron [5]. Iron is essential for bacterial growth and bacteria such as *Escherichia coli *obtain iron from the host by producing siderophores that bind iron and transport it back into the bacterial cell. Studies *in vitro *have demonstrated that Lcn2 has a bacteriostatic effect via the binding of siderophore molecules, thereby restricting the availability of iron to bacteria [5]. Lcn2 mRNA is upregulated markedly following LPS-mediated activation of the toll-like receptor (TLR) 4 signalling pathway [6]. Further supporting a role of Lcn2 as a bacteriostatic agent, mice lacking Lcn2 are markedly more susceptible to bacterial infection compared with wild type mice [6,7]. In all, these observations highlight that Lcn2 is a key participant in the host response where it has the important function to inhibit the growth of bacteria by starving these organisms of essential iron.

While Lcn2 is well accepted to be a secreted protein that binds iron-loaded microbial siderophores, further functions remain unclear. High expression of Lcn2 has been documented in certain organs including the uterus, kidney and mammary glands [8,9]. As such a wide range of cellular functions have been attributed to Lcn2, including a role as an acute phase protein [10], an inducer of tissue involution in reproductive tissues [11], control of kidney tubular cell development [12] and stimulation of apoptosis in hematopoietic cells [8,9]. Recently, Lcn2 was described as a pro-apoptotic factor for murine microglial cells [13] and an autocrine mediator of reactive astrocytosis [14]. Mice with a targeted disruption of the gene for Lcn2 do not show any spontaneous developmental changes nor apoptotic defects [6,7]. Therefore, if Lcn2 has any role in these proposed cellular processes this is clearly redundant. Nevertheless, the discovery of a putative cell surface receptor (termed 24p3R) for Lcn2 on mammalian cells [8], makes it likely that Lcn2 may also modulate cellular function. In support of this, the 24p3R was found to mediate the internalisation of Lcn2 by endocytosis, leading to apoptosis [8]. The 24p3R is widely expressed in different organs [8] but expression in the CNS has so far been little investigated. Overall, the discovery of 24p3R suggests that, in addition to its extracellular anti-microbial function, Lcn2 might have certain intracellular functions, which remain to be defined.

To date the regulation and role of Lcn2 and its receptor in the CNS remains relatively unclear. In this study, the expression and localization of Lcn2 RNA and protein, as well as 24p3R RNA in the CNS were investigated in a previously described model for systemic LPS-induced endotoxemia [15]. In addition, in view of its reported functions as an acute-phase reactant [10] and a modulator glial cell function [13,14] the contribution of Lcn2 to the host response was examined in the CNS following systemic LPS administration.

## Methods

### Animals

Wild type mice (WT; C57BL/6 background) were purchased from the Animal Resources Centre, Canning Vale, WA, AUS. Lcn2 KO mice (C57BL/6 background; [6]) were obtained originally from Dr. Alan Aderem, Institute for Systems Biology, Seattle, USA and a breeding colony was established at the University of Sydney. To match strain background as much as possible, Lcn2 sufficient mice used as controls were derived from the interbreeding of WT mice with Lcn2 KO mice. Lcn2 heterozygotes were then further interbred to derive Lcn2 KO or WT animals which were used in this study. Ethical approval for the use of all mice in this study was obtained from the University of Sydney Animal Care and Ethics Committee.

### Lipopolysaccharide (LPS)-induced endotoxemia model

A previously described model for systemic bacterial infection-induced CNS inflammation was employed in which mice were given either single or dual, staggered injections of LPS [15]. The second dose of LPS mimics a more sustained gram-negative bacteremia in which we have shown previously, provokes a considerably enhanced inflammatory reaction in the brain [15]. In the current study, three-month old mice were injected intraperitoneally (i.p.) with a sub-lethal dose (2 μg/mg body weight) of LPS (from *E. coli *serotype 026:B6; Sigma-Aldrich, Castle Hill, NSW, AUS) in sterile 0.15 M NaCl. Control mice received an i.p. injection of 0.15 M NaCl alone. Mice received either a single injection and were euthanized after 4 h or 24 h, or dual injections given 16 h apart and were euthanized 4 h or 24 h after the second injection.

### Cell culture and treatment

The C8-B4 microglial cell line [16] was obtained from the American Type Culture Collection (Manassas, VA, USA). Cells were cultured in growth medium consisting of Dulbecco's modified Eagle's medium (DMEM; Invitrogen, Mt. Waverley, VIC, AUS) supplemented with 10% FBS (Thermo Scientific, Noble Park, VIC, AUS) and penicillin and streptomycin (Invitrogen). At 75% confluency cells were washed and the medium replaced with fresh DMEM medium containing LPS (*E. coli *serotype 026:B6), polyinosinic:polycytidylic acid (poly I:C) or pertussis toxin (PTX) (all from Sigma-Aldrich) and the cultures incubated for a further 4 h at 37°C before extraction of RNA (see below).

### Isolation of RNA

The brain and liver were removed immediately from euthanized mice, snap frozen in liquid nitrogen and stored at -80°C pending RNA extraction. Total RNA was extracted with TriReagent (Sigma-Aldrich) according to the Manufacturer's protocol. For cells, after removal of treatment medium, cultures were washed once in PBS and RNA prepared using TriReagent performed according to the Manufacturer's protocol.

### RNA Probes

The specific target sequences used to generate probes for murine Lcn2 and the 24p3R for RNase protection assay (RPA) and in situ hybridization (ISH) are listed in Table 1. The generation and sub-cloning of the specific cDNA sequences used as probes were performed by PCR-assisted directional cloning according to our previously published methods [17]. Briefly, cDNA was obtained from 10 μg total RNA (isolated from the brain of mice at 4 h after i.p. injection of LPS as described above) by reverse transcriptase-polymerase chain reaction (RT-PCR) using oligo (dT) primers_12-18 _and 1 μl of Superscript II RT (0.5 μg/μl) as recommended by the manufacturer. The synthesized cDNA was then used as a template and subjected to amplification by PCR. In the PCR, a mixture of 8 μl 10X PCR buffer (without MgCl_2_), 8 μl 50 mM MgCl_2_, 0.5 μl Taq polymerase (5 U/μl), 20 μl RT reaction product from above, together with 5 μl 2 nM 5' primer and 5 μl 2 nM 3' primer in a total volume of 100 μl was prepared. Each cDNA product was then directionally cloned into pGEM-4Z (Promega, Madison, WI, USA) plasmid that incorporate T7 and SP6 RNA polymerase promoters flanking the cloning region defined by the *Eco*RI or *Hind*III restriction enzyme sites. The presence of the correct insert sequence was confirmed by sequencing analysis provided by SUPAMAC (Sydney University Prince Alfred Macromolecular Analysis Centre, Sydney, AUS). Digestion of the transformed plasmids with either *Eco*RI or *Hind*III, transcription with T7 or SP6 RNA polymerase (both from Promega) yielded anti-sense (T7) or sense (SP6) RNA probes respectively. The generation of the additional chemokine, cytokine and host response RPA probe sets used in this study was described previously [18-20]. The genomic clone RPL32-4A served as a probe for the ribosomal protein L32 as described previously [21]. This was included as an internal control for RNA loading during RPA analysis.

**Table 1:** cDNA target sequences used to generate Lcn2 and 24p3R probes

Gene	Target Sequence (bp)	Length (bp)	**GenBank No**.
Lcn2	301-606	305	X14607
24p3R	1449-1554	106	NM_021551
24p3R-ISH	1275-1554	280	NM_021551
RPL32-4A	61 - 139	78	K02060

### RNase protection assay

RPAs were performed as described previously [20] using the probe sets described above consisting of an equimolar pool of EcoRI-linearized templates (15 ng each). RNA levels were quantified from scanned autoradiograms by densitometry using NIH Image software (version 1.63) as described previously [17].

### Dual in situ hybridization and immunohistochemistry

Brains from LPS- or vehicle-injected mice were removed and fixed overnight in ice-cold 4% paraformaldehyde in phosphate-buffered saline (pH 7.4). Paraffin-embedded sections (10 μm) were incubated with [^33^P]-labeled cRNA probes transcribed from either the EcoR1 (antisense) or HindIII (sense) linearized Lcn2 or 24p3R plasmids and processed for in situ hybridization as described previously [20]. Sections were then processed for immunohistochemistry to detect microglia and endothelial cells (biotinylated lectin from *Lycopersicon esculentum*; Sigma-Aldrich), neurons (mouse anti-neuron specific nuclear antigen (NeuN) antibody; Chemicon, Boronia, Vic, AUS), and astrocytes (rabbit anti-cow glial fibrillary acidic protein antibody; DAKO Cytomation; Botany, NSW, AUS). All antibodies were used at a final concentration of 5 μg/ml. Bound antibody was detected using Vectastain ABC kits (Vector Laboratories, Burlingame, CA, USA), and diaminobenzidine/peroxide reagent (Vector) was used as the peroxidase substrate.

### Immunohistochemistry (IHC)

To study the cellular distribution of Lcn2, the astrocyte marker, GFAP or the microglial marker, IBA-1, IHC was performed on paraffin-embedded sections of brain. After deparaffinization in xylene and rehydration in a graded ethanol series, epitope retrieval was performed. For Lcn2 IHC, tissue sections were digested with proteinase K (24 μg/ml; Promega) in 5 × TE (15 min at 37°C) and then washed twice with PBS. For GFAP and IBA-1 IHC, tissue sections were treated as described [22]. Following epitope retrieval, sections for Lcn2 IHC were blocked in PBS plus 10% BSA while sections for GFAP or IBA-1 IHC were blocked in TBS plus 5% goat serum. Sections were then incubated with goat anti-Lcn2 antibody (1:20; R&D Systems, Minneapolis, MN USA), mouse anti-GFAP antibody (1:1000; Sigma-Aldrich), or rabbit anti-IBA-1 antibody (1:100; WAKO, Osaka, Japan) for 1 h at room temperature. After washing (x3) in PBS sections were incubated with biotin-coupled anti-goat, anti-mouse or anti-rabbit secondary antibody (all from Vector) for 40 min at room temperature. Following washing, streptavidin-coupled HRP was applied to the slides for 30 min. Subsequently, specific signals were visualized with NovaRED colour reagent (Vector), counterstained with hematoxylin and the slides examined by bright-field microscopy. To evaluate the specificity of the staining for Lcn2, some sections were incubated either with primary antibody that had been pre-incubated with recombinant Lcn2 in a 20-fold excess or with non-immune goat serum. In addition, immunostaining with the anti-Lcn2 antibody was performed on sections prepared from the brain of LPS-treated Lcn2 KO mice.

### Western Blot Analysis

To investigate the level of Lcn2, transferrin receptor-1 (TFR-1), GFAP or IBA-1 protein, Western blot was employed. Following treatment and euthanasia, the brain was removed and bisected down the sagittal midline and the complete brain half snap frozen in liquid nitrogen. Protein lysates were prepared by homogenizing snap frozen hemibrain in lysis buffer containing 1.5% Nonidet P-40, 10% glycerol, 1 mM DTT (all from Sigma-Aldrich), protease and phosphatase inhibitors (Calbiochem; Kilsyth, Vic, AUS). Protein concentration of the samples was estimated by Bradford Assay (Bio-Rad Laboratories, Gladesville, NSW, AUS) or BCA Protein Assay (Thermo Scientific). An aliquot of sample corresponding to 50 μg/30 μg of protein was separated on a precast NuPAGE gradient gel (4 - 12%; Invitrogen) by electrophoresis at 150 V/40 mA per gel. Following electrotransfer to a PVDF membrane, membranes were blocked with 10% milk/5% milk in 20 mM Tris base, 15 mM NaCl, 0.1% Tween 20 (pH 7.4) (TBS-T). Membranes were then incubated with the primary antibodies: goat anti-Lcn2 (1:1500; R&D Systems), mouse anti-transferrin receptor (TFR-1; 1:5000; Invitrogen), rabbit anti-GFAP (1:5000; Abbiotec, San Diego, CA USA), rabbit anti-IBA-1 (1:2000; WAKO), or mouse anti-GAPDH (1:50000; Merck Millipore, Temecula, CA USA) overnight at 4°C, followed by washing in TBS-T, 1 h blocking in 5% milk in TBS-T and a 1 h incubation with peroxidase-coupled secondary antibody anti goat, anti mouse (1:40000; Sigma-Aldrich) or anti rabbit (1:40000; Santa Cruz Biotechnology Inc., Santa Cruz, CA USA) at room temperature. Bound antibody signal was detected using ECL reagent (GE Healthcare, Castle Hill, NSW, AUS). For quantification of Western blots, densitometric analysis of each band was performed using NIH Image 1.63 software. The density of the individual protein bands were normalized to that of the loading control (GAPDH) and the mean ± S.E.M calculated using Prism 4 software (GraphPad Inc. San Diego, CA USA).

### Statistical analysis

For RPA and Western blot data the statistical significance of any differences was determined either by one-way ANOVA or unpaired, two-tailed t-test as indicated in the figure legend calculated using Prism 4 software (GraphPad). Following ANOVA analysis Tukey's or Dunnette's multiple comparision tests were performed as indicated in the Figure legend. A difference was considered as statistically significant with a p < 0.05.

## Results

### LPS-induced expression of Lcn2 and constitutive expression of the 24p3R genes in the brain

**Figure 1:**
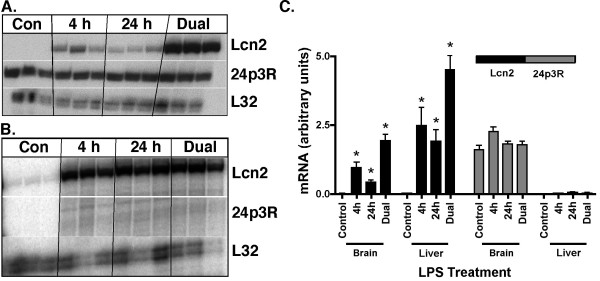
**Lcn2 and 24p3R mRNA levels in the brain and liver following intraperitoneal injection of LPS**. Mice were injected i.p. with sterile 0.15 M NaCl with or without LPS (50 μg) and euthanized after 4 h or 24 h. An extra group of mice received a second LPS injection (dual) 16 h later, and were euthanized 4 h after the second injection. Total RNA was extracted from the brain (A) and the liver (B) and 5 μg analyzed by RPA as described in the Materials and Methods. Quantification of the Lcn2 and 24p3R RNA levels by densitometry (C). For statistical analysis an unpaired, two-tailed t-test was used. For statistical significance * p < 0.05 for LPS- versus vehicle-treated control mice (*p *< 0.05).

To examine the expression of the genes for Lcn2 and 24p3R and determine whether these were regulated in the CNS, a model of bacterial sepsis was employed in which mice received single or staggered, dual i.p. injections of LPS [15]. No Lcn2 mRNA was detectable in the brain (Figure 1A) whereas a low level of Lcn2 mRNA was present in liver of untreated mice (Figure 1B). The liver was included in this experiment as a control organ since strong up-regulation of Lcn2 in the liver following LPS-stimulation has been described previously [6]. After LPS challenge, the level of Lcn2 mRNA increased significantly in both organs (Figure 1A&1B) with a more pronounced increase in the liver (Figure 1B&1C) compared with the brain (Figure 1A&1C). In the liver, the Lcn2 mRNA level was increased 25- and 21-fold at 4 h and 24 h after a single LPS injection, respectively and 46-fold after dual LPS injections (Figure 1C). In the brain, the Lcn2 mRNA level in LPS-treated mice was increased 10- and 5-fold after the 4 h and 24 h single LPS injections, respectively and 21-fold after dual LPS injections (Figure 1C).

In the liver of control or LPS-treated mice 24p3R mRNA was not detectable (Figure 1B). By contrast, strong constitutive expression of 24p3R mRNA was present in the brain of control mice, the level of which was not altered by LPS injection (Figure 1A&1C).

In summary, in normal mice, Lcn2 mRNA was induced to high levels in the liver and brain, following systemic LPS challenge. By contrast, the gene for 24p3R was expressed constitutively in the brain but not in the liver and the mRNA levels for this Lcn2 receptor gene were not altered by LPS challenge.

### LPS-regulated production of Lcn2 protein in the brain

**Figure 2:**
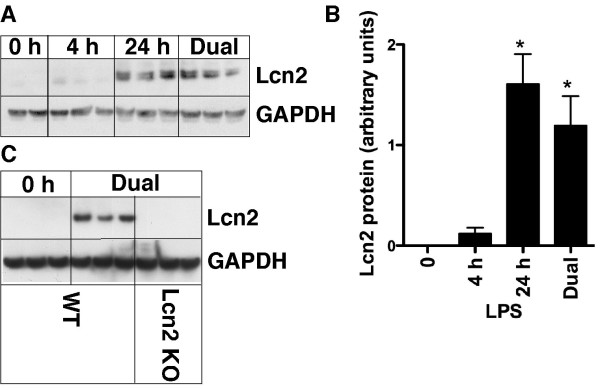
**Lcn2 protein levels in the brain following intraperitoneal injection of LPS**. Mice were treated as shown. Total protein was extracted from the brain and 50 μg analyzed by Western blot (A) as described in the Materials and Methods with quantification (B) of the Lcn2 protein relative to GAPDH loading control. Comparison of Lcn2 protein level by Western blot (C) in brain from WT versus Lcn2 KO mice after dual LPS-treated mice. For statistical significance *, p < 0.05 for LPS-treated mice versus control mice (*p *≤ 0.05).

We next determined if the Lcn2 protein levels in the brain paralleled the changes observed at the RNA level (Figure 2A&2B). By immunoblot analysis, no bands were detectable in lysates of brain from untreated mice. However, at 4 h following LPS injection a weak band was detectable that corresponded with the known size (24 kDa) of Lcn2. The intensity of this band was increased significantly by 24 h after a single LPS injection and was similarly increased to high levels after dual LPS-injections. The specificity of the immunoblot analysis for Lcn2 was confirmed using brain lysates from Lcn2 KO mice (Figure 2C). No bands were detectable in lysates of brain from PBS-treated WT or Lcn2 KO mice. However, following dual injections of LPS a strong band corresponding to Lcn2 was clearly present in lysates of brain from WT but not Lcn2 KO mice (Figure 2C).

These results validated the specificity of the Lcn2 antibody used in our studies and showed that similar to Lcn2 mRNA, Lcn2 protein was markedly induced in a time-dependent fashion in the brain following systemic LPS challenge.

### Differential anatomic localization of Lcn2 and 24p3R RNA in the CNS during LPS-induced endotoxemia

**Figure 3:**
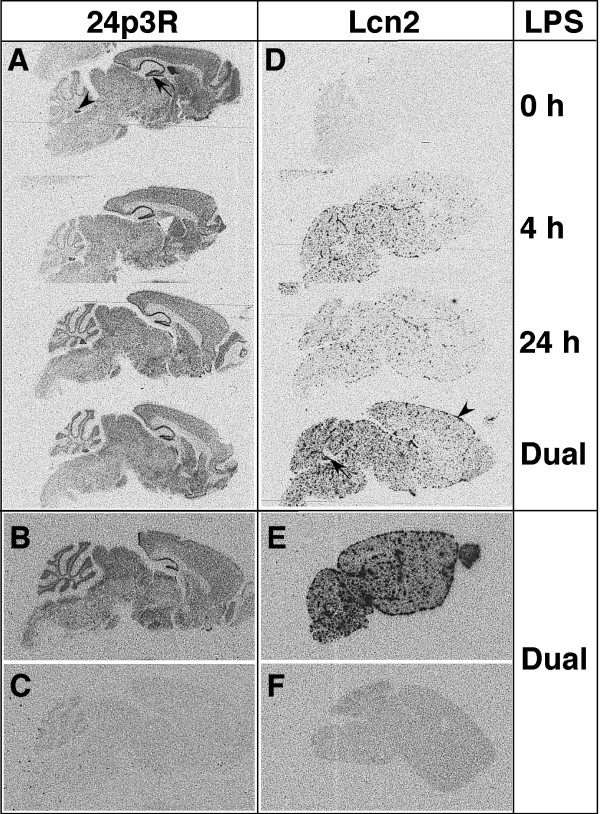
**Anatomical localization of Lcn2 and 24p3R RNA in the brain following intraperitoneal injection of LPS**. Brain was removed from mice at different times following LPS injection, fixed in ice-cold 4% PBS-buffered paraformaldehyde and paraffin-embedded sagittal sections (10 μm) subjected to ISH as described in the Materials and Methods. Hybridization was performed with either 24p3R (A&B) and Lcn2 (D&E) anti-sense or 24pR3 (C) and Lcn2 (F) sense cRNA probes.

To identify the anatomical localization of the Lcn2 and 24p3R RNA transcripts in the brain, in situ hybridization was performed on brain sections from LPS-injected mice using anti-sense and sense RNA probes complementary to the Lcn2 and 24p3R mRNA transcripts (Figure 3).

In parallel with the findings for the RPA, strong 24p3R RNA hybridization signal was detectable at similar levels in 0 h, 4 h, 24 h and dual LPS-injected mice (Figure 3A). The pattern of hybridization signal was distributed widely throughout major gray matter regions of the brain including the cortex and thalamus as well as in various neuronal populations such the granule layer of the cerebellum. However, the highest levels of 24p3R RNA hybridization signal were observed in the dentate gyrus (Figure 3A; arrow) and pyramidal cell layers of the hippocampus and in the choroid plexus (Figure 3A; arrowhead). A comparison of the hybridization of the 24p3R antisense (Figure 3B) versus sense (Figure 3C) RNA probes confirmed that pattern of hybridization seen for the antisense probe was specific.

Comparable with the RPA findings above, little, if any, detectable hybridization signal was seen for Lcn2 RNA in brain from PBS-injected control mice (Figure 3D). By contrast, in brain from 4 h LPS-injected mice, a punctate pattern of Lcn2 RNA hybridization signal was observed evenly distributed throughout the brain parenchyma. At 24 h, the overall pattern of hybridization signal remained similar but was decreased as compared with the 4 h LPS-injected mice. In brain from dual LPS-injected mice, Lcn2 RNA hybridization signal was highest and in addition to expression throughout the parenchyma also included strong expression in the choroid plexus (Figure 3D; arrow) and meninges (Figure 3D; arrowhead). In contrast with dual LPS-treated wild type mice (Figure 3E), no hybridization signal above the sense control levels (not shown) was detectable in brain from Lcn2 KO mice (Figure 3F) following dual injections of LPS.

In summary, these findings confirmed the strong induction of Lcn2 RNA in the brain following LPS treatment and showed that the Lcn2 RNA was localized to cell populations scattered uniformly throughout the brain parenchyma as well as the choroid plexus. By contrast, 24p3R RNA was present at high levels throughout the brain from control and LPS-treated mice and appeared to be localized to mainly various neuronal populations as well as the choroid plexus.

### Distinct cellular localization of Lcn2 and 24p3R RNA in the CNS during LPS-induced endotoxemia

**Figure 4:**
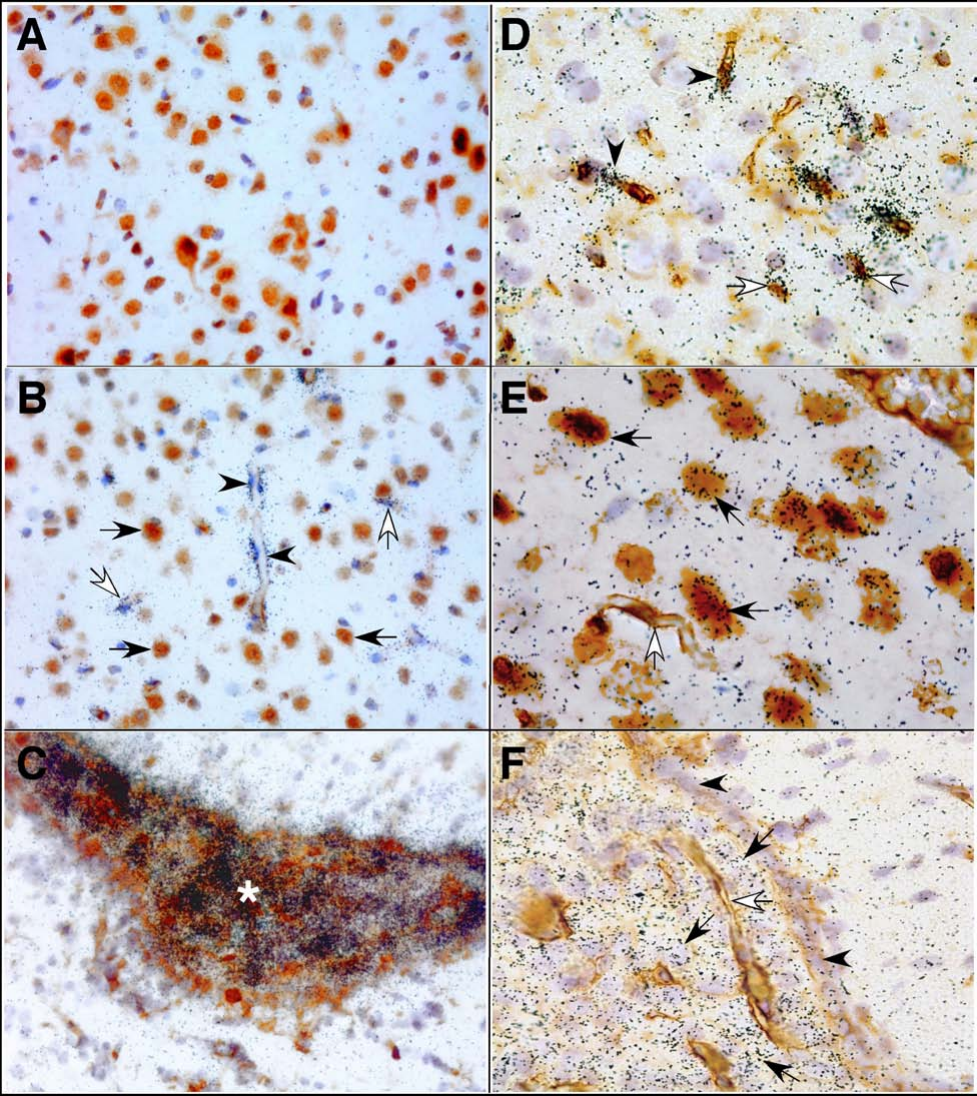
**Cellular localization of Lcn2 and 24p3R RNA in the brain following intraperitoneal injection of LPS**. Paraffin-embedded sagittal sections (10 μm) were subjected to ISH followed by immunohistochemistry as described in the Materials and Methods. A. Brain section from vehicle-injected control mouse hybridized with Lcn2 anti-sense cRNA probe and immunostained for NeuN. B-D. Brain sections from dual LPS-treated mice hybridized with Lcn2 anti-sense cRNA probe and immunostained for NeuN (B) and lectin (C&D). E-F. Brain sections from vehicle-injected control mice hybridised with 24p3R anti-sense cRNA probe and immunostained for NeuN (E) or lectin (F).

To determine the cellular sources of the Lcn2 and 24p3R RNA transcripts, ISH was combined with immunohistochemistry that allowed for the identification of neurons (NeuN), astrocytes (GFAP), microglia (lectin) and endothelium (lectin). No specific signal for Lcn2 RNA was detected above the sense controls in brain from vehicle-injected mice (Figure 4A). In contrast, in brain from mice following dual LPS injections, Lcn2 RNA hybridization signal localized to the vascular endothelium (Figure 4B&4D; arrowheads) as well as some surrounding non-neuronal cells, that, based on nuclear morphology were presumed to be microglia (Figure 4B; white arrows) which was confirmed by lectin stain (Figure 4D; white arrows). The meninges also exhibited Lcn2 RNA hybridization (not shown). The highest level of Lcn2 RNA hybridization signal was observed in the choroid plexus (Figure 4C; asterisk). However, no signal was detectable in neurons (Figure 4B; arrows) or astrocytes (not shown). High Lcn2 RNA hybridization signal was found in cells in close proximity to neurons presumably microglial cells (white arrows) as well as vascular endothelium (black arrows).

In contrast to Lcn2 RNA, 24p3R RNA was localized almost exclusively to neurons in the brain, with a high level in cortical neurons (Figure 4E; black arrows), hippocampal dentate gyrus and granule neurons (data not shown) and Purkinje neurons of the cerebellum (data not shown). Furthermore, a high level of 24p3R RNA was also observed in the choroid plexus (Figure 4F; black arrows). No 24p3R RNA was detectable in astrocytes, microglia or blood vessels (Figure 4E&4F; white arrows). There was no detectable difference in either the level or cellular distribution of the 24p3R RNA expression in brain from vehicle-treated and LPS-treated mice.

In summary, these findings indicated that the cellular localization of Lcn2 RNA and its receptor 24p3R RNA in the brain was on the whole distinct, with the exception of the choroid plexus.

### Localization of Lcn2 protein in the brain of mice with LPS-induced endotoxemia

We next examined the distribution of Lcn2 protein in the CNS by immunohistochemistry. The specificity of the antibody used in these studies was confirmed by the absence of staining (data not shown) in the sections incubated with either non-immune goat serum or after adsorption of the antibody with specific Lcn2 immunizing peptide. In addition, a similar lack of staining was observed in brain sections from Lcn2 KO mice that had been given dual injections of LPS (not shown).

**Figure 5:**
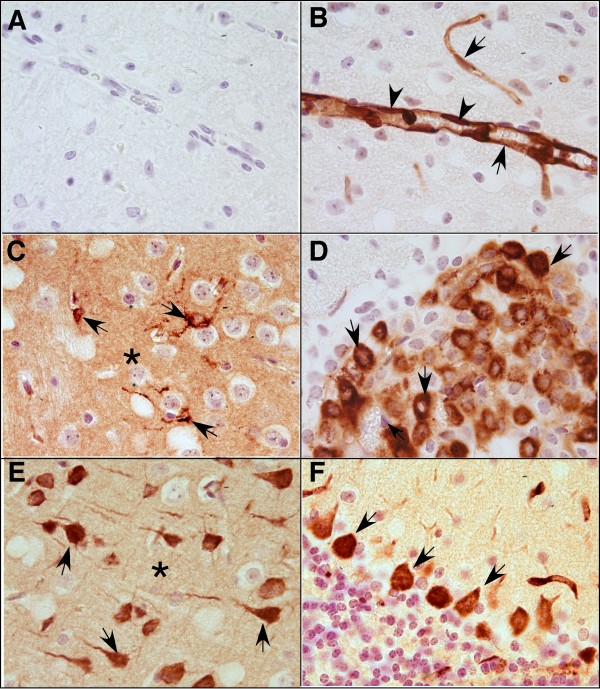
**Cellular localization of Lcn2 protein in the brain following intraperitoneal injection of LPS**. Paraffin embedded sections (5 μm) were stained by immunohistochemistry to detect Lcn2 protein as described in the Materials and Methods. A. Brain section from a PBS-injected control mouse. B-F. Brain sections from mice at 24 h following a single intraperitoneal injection of LPS.

Consistent with the results for the Lcn2 RNA, and similar to the results obtained in LPS-treated Lcn2 KO mice, Lcn2 protein was not detectable in the CNS of control mice (Figure 5A). However, at 4 h after LPS-injection, Lcn2 protein was clearly detectable almost exclusively in endothelial cells in numerous blood vessels throughout the brain (Figure 5B; arrows). In addition to cytoplasmic staining, abundant nuclear staining of the endothelial cells was also evident (Figure 5B; arrowheads). At 24 h after LPS injection, Lcn2 protein accumulated further in vessels throughout the CNS as well as in ramified parenchymal cells identified as microglia (Figure 5C; arrows). In addition, at this time-point there was also strong staining of cells with the morphology and location of epithelial cells in the choroid plexus (Figure 5D; arrows). Unexpectedly, positive staining for Lcn2 protein was observed in specific neuronal populations in the cortex (Figure 5E; arrows), hippocampus (not shown), ventral forebrain (not shown) and Purkinje neurons of the cerebellum (Figure 5F; arrows). Finally, diffuse staining of the parenchyma was evident in various regions of the brain (Figure 5C&5E; asterisk). A similar pattern of staining for Lcn2 protein was observed in the brain from mice following dual LPS injections when compared with 24 h after LPS injection.

Together with the findings from ISH, these results indicated that the predominant Lcn2 producer cells in the CNS of mice with LPS-induced endotoxemia are endothelial cells, choroid plexus-associated cells and microglia. Interestingly, however, there was a marked dichotomy between the ISH and IHC results with regard to various neurons in which Lcn2 RNA was not detectable but Lcn2 protein was clearly present.

### Microglial cells respond to LPS and poly I:C with induction of Lcn2 but lack detectable expression of 24p3R

**Figure 6:**
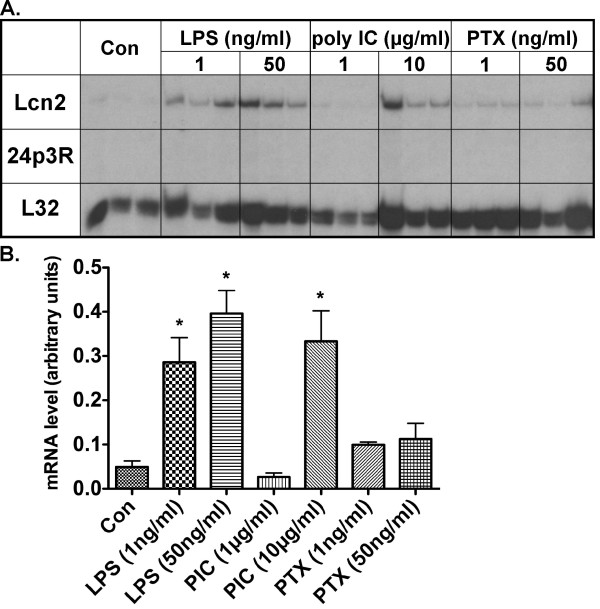
**Regulation of Lcn2 mRNA levels in C8 microglial cells**. The C8 microglial cell line was grown to 75% confluency and treated for 4 h at 37°C as indicted. Total RNA was isolated and analyzed by RPA as described in the Materials and Methods. For statistical analysis one-way ANOVA with Dunnett's multiple comparison test was used. For statistical significance * p < 0.05 versus untreated control (Con).

The findings above indicated that LPS-mediated induction of Lcn2 gene expression by microglia in the brain following systemic injection of LPS. We next examined whether various pathogen associated molecules including LPS, dsRNA (poly I:C) and PTX could directly modulate Lcn2 or 24p3R mRNA levels in cultured C8 microglial cells (Figure 6). A significant dose-dependent increase in Lcn2 mRNA levels was mediated by both LPS and poly I:C. A small increase in Lcn2 mRNA occurred in response to treatment with PTX, however, this was not significant when compared with the vehicle-treated control cells. In contrast to Lcn2, 24p3R was not detectable in C8-microglia cells with or without treatment. These findings showed that in C8 microglial cells, LPS as well as double-stranded RNA is capable of inducing the expression of the Lcn2 gene but these cells appear to lack 24p3R.

### Various markers of the host response to LPS showed similar changes in WT and Lcn2 KO mice

Lcn2 may be an acute-phase reactant [10] and may modulate cellular iron metabolism [8]. In addition, Lcn2 has been suggested to mediate microglial [13] and astroglial [14] activation in response to cytotoxins. Here we employed Lcn2 KO mice to determine if Lcn2 played a role in the cerebral host response to LPS-induced endotoxemia.

**Figure 7:**
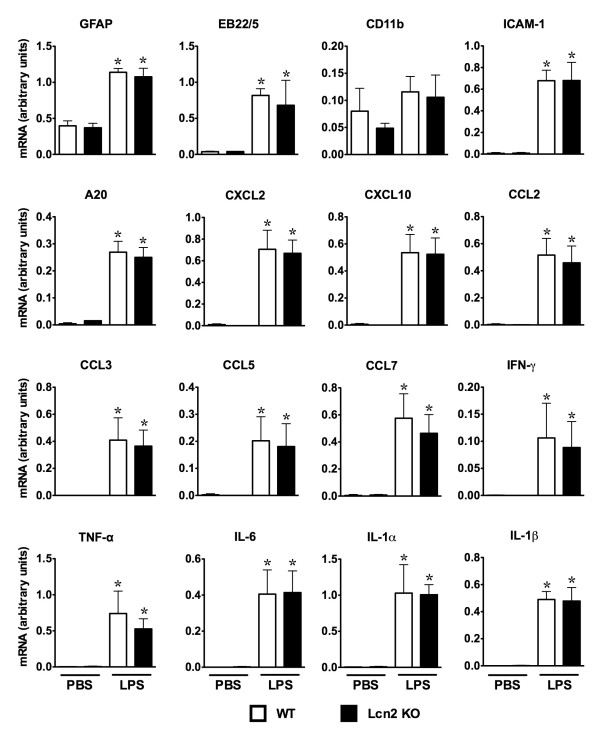
**The level of various host response, chemokine and cytokine mRNA transcripts in the brain of WT versus Lcn2 KO mice following intraperitoneal injection of LPS**. Mice were injected i.p. with sterile PBS with or without LPS and received a second LPS injection (dual) 16 h later and euthanized 4 h after the second injection. Total RNA was extracted from the brain and 10 μg analyzed by RPA as described in the Materials and Methods. For quantification of the mRNA levels, densitometry was performed on film autoradiographs and corrected for loading by reference to the L32 loading control. For statistical analysis, one-way ANOVA with Tukey's multiple comparison test was used. For statistical significance *, p < 0.05 for LPS-treated mice versus vehicle-injected control mice (*p *≤ 0.05).

In brain from WT mice, dual injections of LPS induced a significant increase in the levels of a number of host response (GFAP, EB22/5, CD11b, ICAM-1 and A20), chemokine (CXCL2, CXCL10, CCL2, CCL4, CCL5 and CCL7) and proinflammatory cytokine (IFN-γ, TNF-α, IL-6, IL-1α and IL-1β) mRNA transcripts at 4 h following the second injection of LPS (Figure 7). An almost identical change in the levels of all these mRNA transcripts was observed in similarly treated Lcn2 KO mice. At 24 h following the second LPS injection, with the exception of the Mac1, EB22 and GFAP mRNAs which remained elevated, there was a general decrease in the remaining mRNA transcripts all of which were similarly changed between WT and Lcn2 KO samples (data not shown). These findings suggested that the absence of Lcn2 was not associated with any detectable change in the cerebral host response to systemic LPS injection.

**Figure 8:**
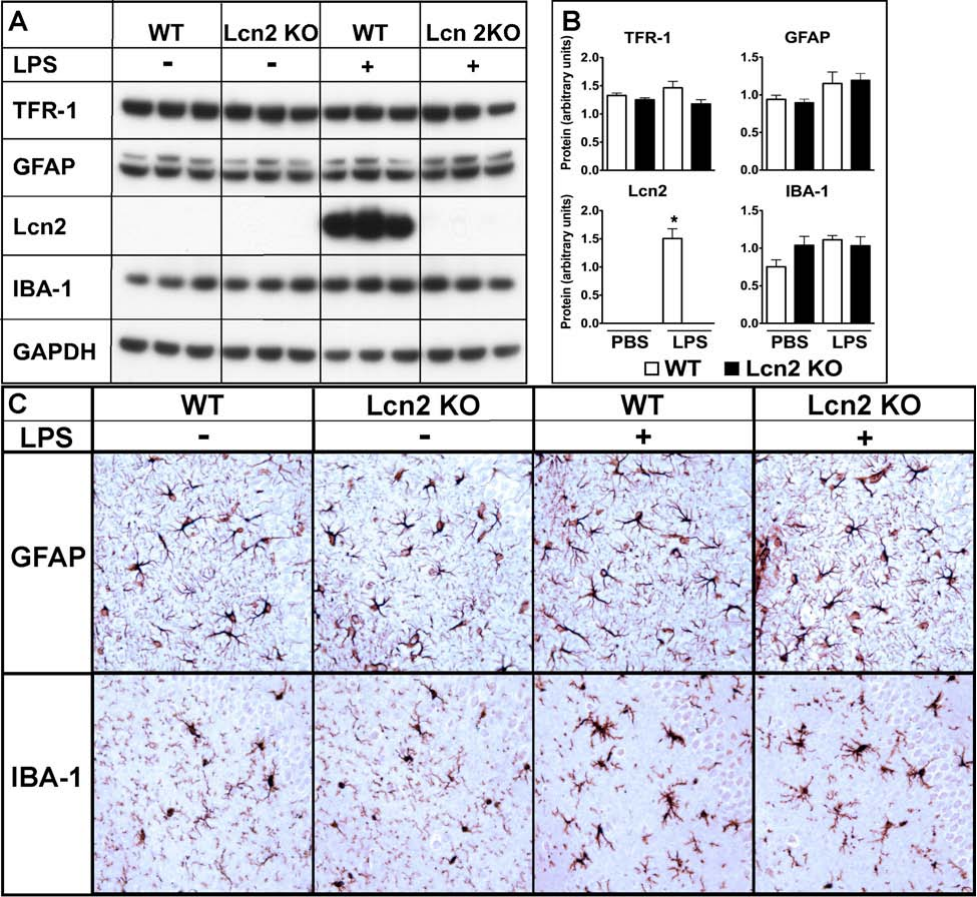
**Comparison of the astrocyte and microglial response in the brain of WT versus Lcn2 KO mice following intraperitoneal injection of LPS**. Mice were injected i.p. with sterile PBS with or without LPS and received a second LPS injection (dual) 16 h later, and were euthanized 4 h after the second injection. Total protein was extracted from the brain and 30 μg analyzed by Western blot (A) and quantified (B) as described in the Materials and Methods. For immunohistochemistry (C) mice were perfused intracardially with ice-cold 4% PBS-buffered paraformaldehyde the brains removed and immunohistochemistry performed for GFAP or IBA-1 as described in the Materials and Methods.

We next examined whether markers for iron handling, the transferrin receptor (TFR-1) and astrocyte (GFAP) and microglial (IBA-1) activation were altered by LPS-induced endotoxemia. In brain from WT and Lcn2 KO mice the level of TFR-1 was similar and remained unaltered at 4 h and 24 h after dual LPS injections. In contrast to GFAP mRNA, which was significantly increased after dual LPS injection (see Figure 7), GFAP protein was not significantly altered from control levels at either 4 h or 24 h (not shown) after dual LPS injections in either WT or Lcn2 KO mice (Figure 8A &8B). Similar to GFAP, the level of the microglial marker IBA-1 did not differ significantly between WT and Lcn2 KO mice and remained unaltered following LPS injection in either WT or Lcn2 KO mice (Figure 8A &8B). To determine further whether Lcn2 may contribute to glial activation in response to LPS-induced endotoxemia we performed immunohistochemical analysis of brain sections. Immunostaining for GFAP (Figure 8C) revealed similar morphological appearance and GFAP levels in astrocytes from WT and Lcn2 KO mice. Little observable change was found for these parameters in either WT or Lcn2 KO mice at 4 h or 24 h (not shown) after LPS injection. Both IBA-1 staining and microglial morphology was similar for WT and Lcn2 KO mice in which the cells exhibited a highly ramified appearance with fine processes that extended throughout the parenchyma of the brain (Figure 8C). However, following dual LPS injections the microglia underwent a marked morphological change in which the processes appeared retracted and were more stunted and the cells more swollen in appearance in both WT and Lcn2 KO mice (Figure 8C). In total, these data failed to find any significant difference in the astroglial nor microglial response to LPS between WT and Lcn2 KO mice.

## Discussion

Lcn2 has emerged as a key factor in the host response to bacterial infection [3]. Moreover, based on recent reports of cellular receptors that mediate the binding and uptake of Lcn2 it is likely that Lcn2 may also influence the function of eukaryotic cells [8,23,24]. Currently little is known concerning the CNS pathobiology of Lcn2 or its putative cellular receptor 24p3R [8]. Therefore, here we investigated the expression and localization of Lcn2 RNA and protein, as well as 24p3R RNA, in the brain in a model for systemic LPS-induced endotoxemia. Similar to the liver, we found that Lcn2 mRNA and protein, although not detectable in the brain of normal control mice, were induced by 4 h after a systemic LPS challenge and increased markedly following a repeated challenge with the inflammogen. The dynamics of Lcn2 induction in the brain following LPS administration suggests that in this tissue as with the liver [6,10], Lcn2 functions as an acute-phase response protein. Consistent with its role as an acute phase reactant, Lcn2 is secreted from various cells including microglia [13] and accumulates to high levels in the blood following intraperitoneal injection of LPS in mice [6]. In addition, Lcn2 was shown to increase in the cerebrospinal fluid of LPS-treated mice [25]. Despite a significant reduction in Lcn2 mRNA levels, the increased accumulation of Lcn2 protein we observed in the brain at 24 h after LPS treatment suggested that Lcn2 protein is also relatively stable and may undergo limited turnover in the brain. In contrast to the ligand, we demonstrated that there is high constitutive expression of 24p3R in the brain of mice, which was not affected by systemic LPS administration. In line with our finding here of CNS and predominant neuronal localization, DNA sequence analysis revealed that 24p3R corresponds to the brain type organic cation transporter [8]. Although it has been reported previously that the 24p3R protein is present in the liver [8], we failed to detect the corresponding 24p3R mRNA. Therefore, our finding suggests that unlike the brain, the 24p3R gene is not expressed in the liver.

A previous study [25] reported that Lcn2 protein accumulates in the choroid plexus of mice following systemic LPS injection. Our findings here confirm and extend these observations and illustrated further the induction of high levels of Lcn2 mRNA in choroid plexus epithelium indicating that this brain tissue is a major CNS site for the production of Lcn2. However, the induction of significant Lcn2 protein levels in the choroid plexus occurred relatively late after LPS administration. The earliest detectable induction of Lcn2 mRNA and protein following LPS injection was in the cerebrovascular endothelium which increased with time. This vascular site of production likely leads to the initial accumulation of Lcn2 at the blood-brain barrier interface. Subsequent passage of LPS across the fenestrated vascular endothelium of the choroid plexus could account for Lcn2 production at this site, leading to the additional accumulation of Lcn2 at the blood-CSF barrier. We observed more extensive induction of Lcn2 mRNA, which included parenchymal microglia throughout the brain following dual injections of LPS. Our studies revealed that LPS is capable of directly inducing the production of Lcn2 by the C8 microglial cell line *in vitro*. The ability of LPS to activate the expression of the gene for Lcn2 in mice is crucially dependent on TLR4 signaling [6]. The known pattern of TLR4 expression in the CNS of mice is consistent with a role for TLR4 in mediating LPS-induced Lcn2 expression in the choroid plexus, blood vessels and microglia [26,27]. The finding that poly I:C, an activator of TLR3 and RNA helicase signaling [28] also induced Lcn2 gene expression in the C8 microglia indicates that additional innate signaling pathways associated with viral infection may also regulate the expression of the Lcn2 gene.

Our studies revealed a major discrepancy in the cellular localization of the Lcn2 RNA versus protein in the brain following LPS injection. While neurons did not contain detectable levels of Lcn2 RNA, subsets of these cells in different regions of the brain stained strongly for Lcn2 protein. The specificity of the anti-Lcn2 antibody used in our studies was confirmed via a number of approaches making it unlikely that this neuronal staining was non-specific or an artifact. It is possible that Lcn2 RNA was present but at a very low level in neurons that was undetectable by in situ hybridization histochemistry. An alternative and perhaps more plausible explanation for these findings was that neurons can actively take up and accumulate Lcn2 that was secreted by microglia. The finding of diffuse immunostaining for Lcn2 on the neuropil suggested that relatively high levels of Lcn2 may accumulate in the extracellular milieu following systemic LPS administration and therefore would be available for cellular uptake. We showed that neurons had high levels of expression of the 24p3R gene which encodes for a protein that is known to facilitate the binding and cellular uptake of Lcn2 [8]. However, the distribution of the 24p3R RNA was quite widespread and included the majority of neurons in the brain whereas Lcn2 immunostaining was restricted to specific sub-populations of neurons in different brain regions. The basis for the restricted localization of Lcn2 to specific neurons is unknown but raises questions about the overall role for 24p3R in this proposed process. Recently, megalin (also known as lipoprotein receptor-related protein-2) was identified as another putative cellular receptor that supports the uptake of Lcn2 by human cells [23]. However, it is unclear presently what the cellular distribution of this putative Lcn2 receptor is in the brain. Irrespective of the explanation, our findings raise the strong possibility that neurons may be a target for the actions of Lcn2 in the brain.

The robust induction of Lcn2 by LPS and other inflammatory stimuli and the linkage of this protein to the acute-phase response led us to examine various parameters of the host response to LPS in the brain in mice deficient for Lcn2. Our findings here extend those of a previous study [15] in demonstrating the dramatic induction of various genes associated with the host response to staggered peripheral injections of LPS. However, the findings also suggested that Lcn2 does not play a major role in this process since similar changes were observed in the level of expression of various cytokine and chemokine genes as well as the GFAP, CD11b and EB22 genes in both WT and Lcn2 KO mice following LPS treatment. Previous studies have suggested that Lcn2 may sensitize microglia to nitric oxide-induced apoptosis and induce morphological changes consistent with activation [13]. Moreover, subsequent work from the same group [14] reported a similar outcome in astrocytes i.e. Lcn2 sensitized these cells to cytotoxin-induced cell death and mediated astrocytosis. However, the current study indicated that there was no significant change in either microglial or astrocyte activation in the brain of Lcn2 KO mice in response to peripheral LPS treatment. Therefore, our findings suggest that Lcn2 is not an essential modulator of gliosis in response to LPS *in vivo*.

## Conclusions

Based on the findings from this study, we conclude: 1) Lcn2 production is strongly induced in the CNS by systemic LPS injection, 2) in addition to Lcn2 production at key gateways of bacterial entry to the CNS, neurons may be a target for the actions of Lcn2, which is apparently taken up by these cells and 3) the cellular functions of Lcn2 in the CNS response to systemic LPS challenge remain unclear and will require further study to elucidate.

## List of abbreviations

CNS: central nervous system; Lcn2: lipocalin 2; LPS: lipopolysaccharide; TLR: toll-like receptor; WT: wild type.

## Competing interests

The authors declare that they have no competing interests.

## Authors' contributions

JPKI and ALN performed research experimentation, data and statistical analysis and writing of the manuscript. MJH, SLL and MM contributed to the research experimentation and assisted in manuscript preparation. ILC was responsible for the design and performance of the research project and drafted and submitted the manuscript for publication. All authors read and approved the final manuscript.
